# Smartphone GNSS Performance in an Urban Scenario with RAIM Application

**DOI:** 10.3390/s22030786

**Published:** 2022-01-20

**Authors:** Antonio Angrisano, Salvatore Gaglione

**Affiliations:** 1 Department of Engineering, University of Messina, Contrada di Dio, Sant’Agata, 98166 Messina, Italy; 2 Department of Science and Technology, University of Naples “Parthenope”, Centro Direzionale Isola C4, 80143 Naples, Italy; salvatore.gaglione@uniparthenope.it

**Keywords:** smartphone GNSS, RAIM, mass-market devices, urban scenario

## Abstract

In an urban scenario, GNSS performance is strongly influenced by gross errors in the measurements, usually related to multipath and non-line-of-sight phenomena. The use of RAIM algorithms is a common approach to solve this issue. A significant amount of the existing GNSS receivers is currently mounted on smart devices, above all, smartphones. A typical drawback of these devices is the unavailability of raw measurements, which does not allow fully exploiting the GNSS potential; in particular, this feature limits the use of RAIM algorithms. Since 2016, for few smart devices, it has been finally possible to access GNSS raw measurements, allowing the implementation of specific algorithms and enabling new services. The Xiaomi Mi 8 is equipped with the Broadcom BCM47755 receiver, able to provide dual-frequency raw measurements from quad-constellation GPS, Glonass, Galileo, BeiDou. In this work, the performance in an urban area of the Xiaomi Mi8 GNSS was analyzed. An important issue of smartphone GNSS is related to the antenna, which is not able to protect from the multipath phenomenon; this issue has a large probability to emerge in hostile environments like urban areas. As a term of comparison, the high-sensitivity receiver NVS NV08C-CSM, connected to a patch antenna, was used. In particular, the considered receivers were placed in the same location, and their positions were estimated in single point positioning, applying a classical RAIM algorithm. An error analysis was carried out, and the obtained results demonstrated the effectiveness of RAIM when applied to Xiaomi Mi8 GNSS measurements.

## 1. Introduction

Currently, a large amount of GNSS receivers is installed into smart devices. According to the GNSS Market Report from the European GNSS Agency [1], in 2019, 1.8 billion of GNSS receivers were sold; about 1.6 billion of units were mass-market devices, costing less than 5 euros, and about 90% of these were mounted on smartphones or wearables (smart watches, fitness trackers, smart glasses). In 2020, the number of GNSS devices in use is almost 7 billion; about 6 billion are in the consumer solution market, and about 3.5 billion of these are into smartphones.

A typical drawback of the smartphone GNSS is the unavailability of raw measurements, which does not allow fully exploiting the device capability. Indeed, without accessing the raw measurements, it is only possible to use the position provided by the chip manufacturer as it is.

In May 2016, Google announced the possibility to retrieve GNSS raw measurements from Android 7 smart devices. This technical development has opened the door to a wide range of possible applications for smartphones, based on advanced GNSS processing techniques, typically restricted to professional receivers. For instance, thanks to raw measurements availability, code-aided positioning and differential positioning or PPP (Precise Point Positioning) techniques could be used in smartphones, improving location-based services. In addition, RAIM (Receiver Autonomous Integrity Monitoring) algorithms could be finally implemented, allowing the real-time analysis of position reliability. The performance assessment of RAIM, applied to the smartphone, is the main scope of this work.

The scientific community has immediately demonstrated interest in this opportunity; indeed, since 2017, GSA has coordinated a “Task force for GNSS raw measurements” and in 2017, it published a white paper, describing the details of measurements retrieving and the potentialities of their use [2].

In [2], the results of a kinematic test are shown; the adopted equipment included a Nexus 9 tablet and a low-cost single-frequency receiver connected to a geodetic antenna; a code positioning was performed. The experiment took place in two scenarios: open sky and an urban area. The performance of the considered devices was similar in the open sky, but that of Nexus evidently degraded in the urban area. This was due to the linearly polarized antenna of Nexus, not able to remove a multipath. Hence, a GNSS chip in a smart device can provide a performance comparable to that of a low-cost GNSS receiver, if no multipath is present.

Before the announcement of raw measurements availability with Android 7, this issue was just emphasized in some pioneering research about smartphone GNSS chips, carried out thanks to custom hardware and/or software solutions. In [3], the performances of a smartphone antenna, a patch antenna, and a geodetic antenna were compared. The smartphone antenna’s poor multipath suppression and irregular gain pattern were identified as an impediment to precision positioning. In [4], the performances of Nokia Lumia 1520 smartphone, u-blox mass-market receiver, and a Novatel geodetic receiver were compared; the measurements were processed in single-base and network RTK mode, exploiting the Finnish national GNSS network. The results demonstrated that smartphone measurements are less precise with respect to those of the other receivers, and frequent outliers are present. To access the raw GNSS measurements, a custom firmware, courtesy of Microsoft, was used. In [5], the feasibility of precise positioning with a smartphone was assessed. Samsung S5 device was tested, and the multipath phenomenon was indicated as the main obstacle to centimeter positioning. A test phone with customized software was used to obtain raw measurements. 

After the Google announcement, several researchers analyzed the performance of smartphone GNSS chips from different points of view. A study [6] analyzed the behavior of Nexus 9 tablet GNSS measurements, compared with a Novatel geodetic receiver, in a benign environment; from the experiment, it was clear that Nexus measurements were noisy and prone to multipath. Nexus C/N0 values were significantly lower (about 10 dB-Hz) and more variable with respect to Novatel values. The performance of the GNSS chip in Huawei Mate9 smartphone were evaluated in [7]; different antenna configurations were used: internal smartphone antenna, external helical antenna, and external pinwheel antenna. Test were carried out in open sky and urban canyon scenarios. The results demonstrated that the GNSS smartphone performance could be significantly enhanced by connecting an external antenna. The performance of several smartphone GNSS chips, in particular Samsung S8 and S8+, Huawei Mate 9 and Mate 10, and Xiaomi Mi8, were analyzed in [8]. Samsung and Huawei devices are single-frequency; Xiaomi is double-frequency, being able to receive L1/L5 signals. All the considered receivers are multi-constellation. From the experiments in open sky, the authors inferred that Galileo and BeiDou pseudorange measurements were more precise than the GPS and Glonass ones, and Xiaomi chip outperformed the single-frequency devices. Xiaomi Mi8 performance was evaluated in open sky and urban area in [9]; a geodetic receiver was used as a term of comparison. The main purpose of the work was to study the code multipath phenomenon, and the authors concluded that the smartphone performance in terms of multipath was far from that of the geodetic receiver. The PPP performances of Huawei Honor 9, Huawei P10, and Xiaomi Mi8 were compared in [10], highlighting the best quality of the Xiaomi Mi8 measurements with respect to the ordinary single-frequency smartphones. In [11], a test in a sub-urban environment was conducted on Xiaomi Mi8 receiver, processing the measurements with the RTK and PPP techniques and obtaining errors of few meters. The performances of Samsung Galaxy S8+ and Huawei P10 Plus GNSS chips in NRTK techniques were tested in [12]; the results were compared with those of a u-blox mass-market receiver. Both smartphones were able to reach meter-level accuracy, while cm-level accuracy was obtained with the u-blox. In [13], the double-frequency GNSS chip of Xiaomi Mi8 with PPP technique was tested in benign environments, obtaining decimeter-level accuracy in static mode and few-meter level accuracy in kinematic mode. In [14], raw measurements from several devices, including smartphone chips, low-cost, and high-grade receivers were processed in PPP mode. Among the considered smartphones, there were the double-frequencies Xiaomi Mi8 and Huawei Mate 20. In open sky, the convergence period and the position accuracy of dual-frequency smart devices were similar to those of high-grade receivers; high values of pseudorange and carrier-phase residuals indicated the presence of an unmodeled hardware bias and high multipath and noise, for the smartphones. In kinematic mode, the smartphone accuracy was of few meters, significantly worse with respect to that of low-cost receiver.

From the literature, it is clear that the main issue of smartphone GNSS is related to the antenna, which is not able to protect from the multipath phenomenon; the issue has large probability to emerge in hostile environments like urban areas. Few research studies on smartphone GNSS chips included tests in signal-degraded areas ([9,11,14]), and the application of RAIM algorithms has not been taken into account.

In this work, the performance of Xiaomi Mi8 GNSS in an urban scenario was analyzed. The high-sensitivity receiver NVS NV08C-CSM was used as a term of comparison. In an urban scenario, the presence of blunders among the measurements is very common; hence, a classical RAIM algorithm was applied. From the obtained results, the benefit of using RAIM was evident for Xiaomi Mi8 receiver. The applied approach is valid for any kind of smartphone GNSS, because of the characteristics of their antennas, which are not able to protect from the multipath phenomenon. The positioning technique adopted in this research, that is the code positioning, is explained in Section 2. RAIM concept and subset testing algorithm, used in this work, are described in Section 3. The test carried out is described in Section 4, while the results are shown in Section 5; finally, the conclusions are drawn in the last section.

## 2. Absolute Positioning with Pseudorange Measurements

Absolute positioning with pseudorange measurements, also called Single Point Positioning (SPP), is the basic GNSS mode; it is based merely on pseudorange observations, PR, whose equation is

(1) PR=ρ+b+ε

Here, ρ is the geometric range between receiver and satellite, b is the receiver clock offset in distance unit, the term ε includes errors not corrected by models (orbital, multipath, noise) and residuals of the partially corrected errors (satellite clock, relativistic, ionospheric, tropospheric). The unknown parameters of the problem are the receiver coordinates (embedded in ρ) and the offset b.

Equation (1) is linearized around a set of approximate unknown parameters; a set of m linearized equations in matrix form is

(2) $$\underline{z}=H\underline{\Delta x}+\underline{\varepsilon }$$

$\;\underline{z}$ is the measurement vector, containing the difference between actual and computed pseudoranges, H is the design matrix, $\underline{\varepsilon }$ contains the un-modelled and the residual errors, $\underline{\Delta x}$ contains the unknowns of the linearized equation.

The vector $\underline{\Delta x}$ can be obtained with an estimation technique; usually, the weighted least squares (WLS) or the Kalman filter are adopted.

**Figure 1:**
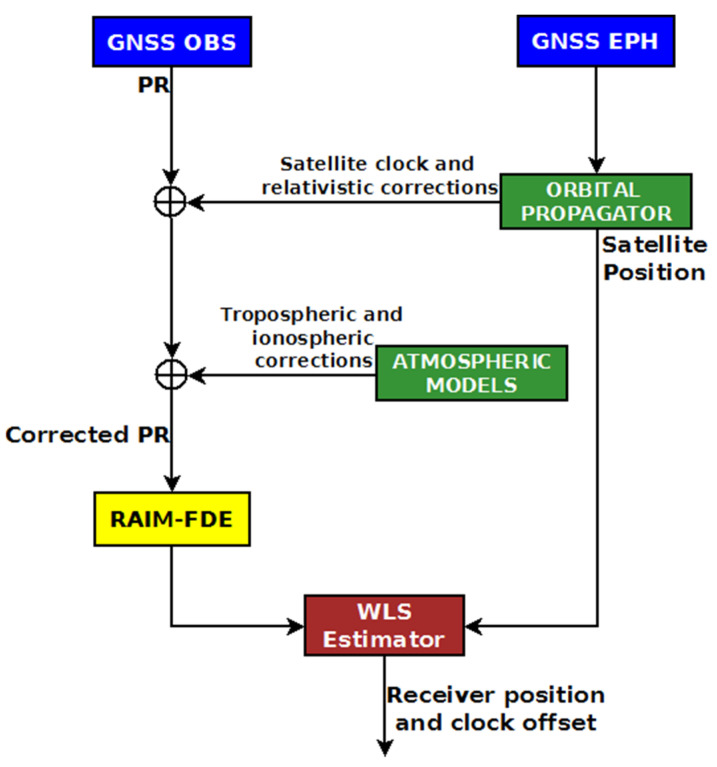
Scheme of absolute positioning with pseudorange measurements (adapted from [15]).

In Figure 1, a scheme describing the SPP algorithm is shown. In the scheme, a RAIM-FDE (Fault Detection and Exclusion) block is included too. The main inputs of SPP are the GNSS observables, in particular, the pseudoranges, and the GNSS ephemerides, from the navigation message. A suitable algorithm (in the “orbital propagator” block) computes the satellite positions and clock errors at the transmission epochs. The measured pseudoranges are corrected for the atmospheric, relativistic, satellite clock errors and are processed by the WLS estimator to obtain receiver position and clock error. The RAIM-FDE block detects and rejects the anomalous measurements.

## 3. RAIM

RAIM is a GNSS augmentation technique, whose purpose is monitoring the integrity of the GNSS solution. RAIM has been developed to meet the integrity requirements of civil aviation [16,17,18]. The main tasks of RAIM are

- detecting the presence of anomalous measurements, eventually rejecting them for solution estimation, and
- computing the protection levels, a statistics bound to position error.

RAIM-FD (Fault Detection) configuration is simply able to detect the presence of anomalous measurements, often referred to as blunders or outliers; RAIM-FDE is also able to identify and reject the outliers.

RAIM has been successfully adapted to work in signal-degraded environments, such as urban areas, with the purpose of reducing the effects of frequent blunders [15,19,20,21].

RAIM core is a consistency check on redundant measurements, usually based on residuals analysis [22]. The residuals are defined as the difference between actual and predicted measurements

(3) $$\underline{r}=\underline{z}-H\hat{\underline{\Delta x}}$$

The residuals are related to measurement errors $\underline{\varepsilon }$; in general, low residuals values indicate low measurement errors and consistent measurements.

A common RAIM algorithm, often referred to in the literature as “global test”, uses a quadratic form of the residuals to define a decision variable D:

(4) $$D={\underline{r}}^{T}W\underline{r}$$

where W is the weighting matrix, defined as the inverse of the measurement error variance–covariance matrix.

D is compared with a threshold T, whose value depends on the required RAIM performance and on the assumed behavior of D, in turn related to the assumed behavior of $\underline{\varepsilon }$ [16,23].

If D exceeds T, the presence of an outlier is supposed and, in the case of the RAIM-FD algorithm, the GNSS solution is considered unreliable. On the other hand, RAIM-FDE algorithms try to identify and reject the outliers. A common FDE scheme is the “observation subset testing” [15,20] which iteratively applies the global test to all the possible combinations of the available measurements to identify a blunder-free subset, if it exists.

## 4. Test

**Figure 2:**
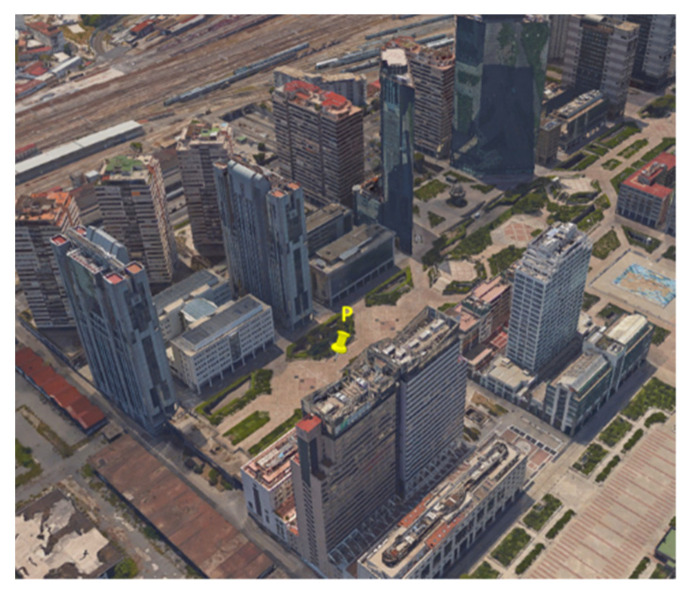
Scenario of the test at the “Centro Direzionale”, Naples, Italy.

In order to pursue the goal of the work, a data collection was carried out in an urban scenario; the location of the test was the district “Centro Direzionale” in Naples (Italy), characterized by several skyscrapers which block and reflect GNSS signals. The considered devices were a smartphone Xiaomi Mi8 and an NVS receiver NV08C-CSM. The scenario of the test is shown in Figure 2; the devices were placed in point P in the figure.

Xiaomi Mi8 is equipped with the Broadcom BCM47755 GNSS chip, able to receive GPS, Glonass, Galileo, BeiDou signals on dual-frequencies L1 and L5. The app Geo++ is used to retrieve raw measurements from Xiaomi Mi8.

NVS NV08C-CSM is a high-sensitivity receiver able to receive multi-constellation signals on L1 frequency. The particular device, used in this experiment, is able to receiver only GPS and Glonass signals, so the two devices were compared only in terms of the common constellations, i.e., GPS and Glonass. The NVS receiver was connected to a low-cost patch antenna.

**Figure 3:**
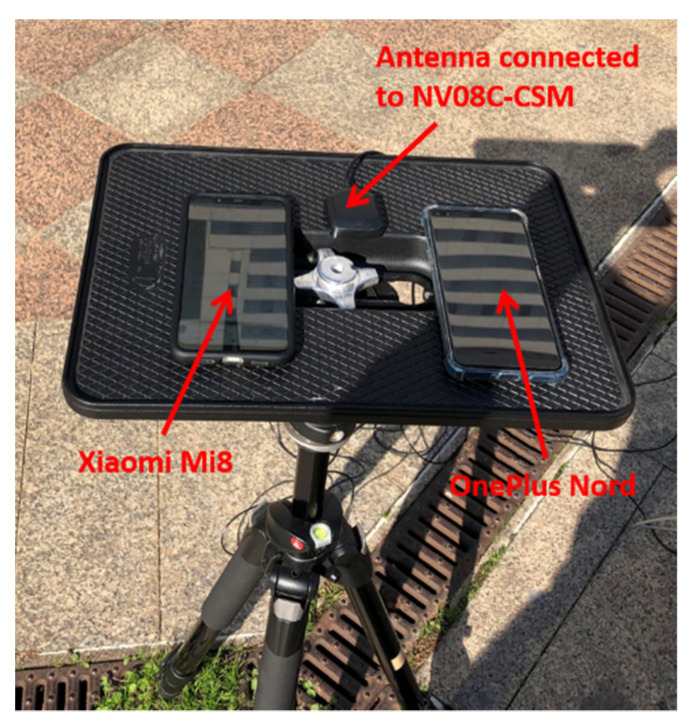
Devices used in the experiment.

The considered devices were put on a small platform, placed at a known position, whose coordinates were determined with mm accuracy by topographical techniques. In Figure 3, the platform is shown. The antenna connected to the NVS receiver and the smartphone Xiaomi Mi8 were placed only few cm apart. Another smartphone, the OnePlus Nord, was placed on the same platform and was used to collect GNSS measurements; unfortunately, the raw measurements from the OnePlus device demonstrated an anomalous behavior and were unusable for positioning. For this reason, the results from OnePlus Nord are not discussed in this paper.

The data were collected on 17 September 2020, for about 1 h, and the stored measurements were processed by the PANG-NAV tool [23] with and without RAIM-FDE functionality. The obtained positions were compared with the ground truth, carrying out an error analysis. A mask angle of 5° and a C/N0 threshold of 20 dB-Hz were applied for the processing.

## 5. Results and Discussion

In order to compare the performances of Xiaomi Mi8 and NVS GNSS devices, several analysis were carried out:

- Analysis of the satellite visibility and geometry,
- Analysis of the C/N0 values for the received signals,
- Analysis of the position performance, without applying RAIM functionality,
- Analysis of the position performance, applying RAIM functionality.

### 5.1. Analysis of the Satellite Visibility and Geometry

Despite the devices being placed in the same location, it was useful to study the actual number of used GNSS signals, which strongly affected the computed position. In Table 1, the minimum, average and maximum number of available satellites, for both considered devices and constellations, are shown. It is evident that a larger number of satellites was available for NVS; this was valid for both GPS and Glonass systems. In particular, the maximum number of satellites was similar for NVS and Xiaomi Mi8, but a larger gap was present in the minimum and average values. This phenomenon is probably related to the different grades of the used antennas; the Xiaomi Mi8 antenna, such as the smartphone antennas in general, is less sensitive compared with a low-cost patch antenna [3].

The observed satellite geometry is related to the number of available satellites; indeed the DOP (Dilution Of Precision) parameters decrease with the number of satellites. In Table 2, a comparison between PDOP (Position DOP) values for Xiaomi Mi8 and NVS is shown. In accordance with satellite visibility, the PDOP values of NVS were significantly lower than those of Mi8. The performance gap was evident mainly on maximum PDOP values with GPS and GPS/Glonass constellations: with GPS only, the maximum PDOP values were 16.6 for Xiaomi Mi8 and 5.6 for NVS, while with GPS/Glonass, the maximum PDOP values were 10.9 for Xiaomi Mi8 and 2.9 for NVS.

### 5.2. Analysis of the C/N0 Values for the Received Signals

The C/N0 parameter is an indicator of the quality of the GNSS signals and, consequently, of the measurements. In Table 3, a comparison between the C/N0 values for Xiaomi Mi8 and NVS is shown. It is evident that the quality of the Xiaomi measurements, according to the C/N0 values, was significantly worse than that of NVS. Indeed, for GPS measurements, the average and maximum C/N0 values were 33.2 and 43.0 for Xiaomi and 42.7 and 55.0 for NVS, respectively. The Glonass measurements were characterized by lower C/N0 values with respect to the GPS ones, with higher values for NVS with respect to Xiaomi.

**Figure 4:**
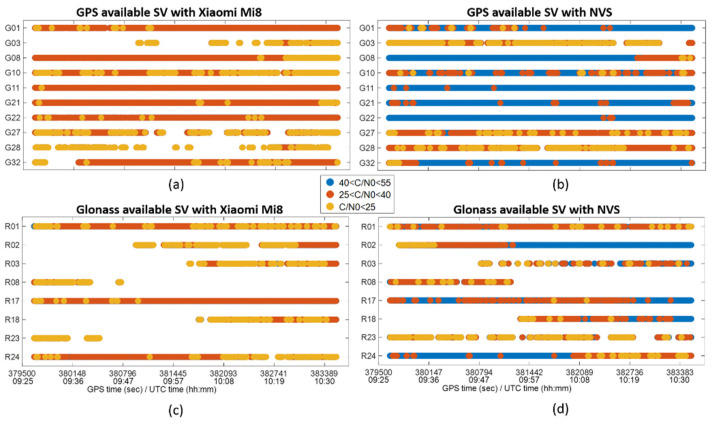
Available satellites and C/N0. Frame (**a**) refers to GPS satellites available from Xiaomi Mi8. Frame (**b**) refers to GPS satellites available from NVS. Frame (**c**) refers to Glonass satellites available from Xiaomi Mi8. Frame (**d**) refers to Glonass satellites available from NVS.

In Figure 4, the available satellites during the session for Xiaomi Mi8 and NVS are shown. Satellite availability is indicated by a colored dot, representing the quality of the measurement in terms of C/N0; in details, values of C/N0 between 40 and 55 dB-Hz are in blue, those between 25 and 40 in red, those under 25 in yellow. Frames (a) and (c) display, respectively, GPS and Glonass situations with Xiaomi Mi8, while frames (b) and (d) show GPS and Glonass situations with NVS. The set of satellites seen at least one time during the session was the same for both devices, but for Xiaomi, the C/N0 values were evidently lower, and several discontinuities were present. For instance, for NVS, satellite G27 (frame (b)) had prevalently medium C/N0 values (red), rarely high values (blue), and often low values (yellow); for Xiaomi, the same satellite (frame (a)) was characterized by prevalently low C/N0 values and by some evident tracking discontinuities.

### 5.3. Analysis of Position Performance, without Applying RAIM Functionality

In order to assess the performance of Xiaomi Mi8 and NVS GNSS devices, a comparison in position domain was carried out. The location of the test was known, and the positions of the devices were estimated in single point mode, using the PANG-NAV tool [23], with and without RAIM functionality. The considered figure of merits for the comparison were RMS (root-mean-square) and maximum errors, both horizontal and vertical.

In Table 4, the processing results without RAIM application are shown. About GPS-only solutions, Mi8 showed smaller RMS errors than NVS, probably because a larger number of blunders were present among the NVS measurements. Conversely, in Glonass-only solutions, NVS showed smaller RMS errors, because the Glonass visibility and geometry for Mi8 were often very poor, causing bad positioning. In the GPS/Glonass case, the NVS horizontal RMS error was slightly smaller with respect to Mi8 error, while Mi8 vertical RMS error was clearly smaller than the NVS one. When analyzing the maximum errors, it was evident that NVS performance prevailed on Mi8 performance Finally, the last column in Table 4 shows the “solution availability”, defined as the percentage of epochs in which the solution is available, i.e., when there is a sufficient number of measurements for solution computation. Mi8 and NVS solution availability was similar in the GPS and GPS/Glonass cases, while it was significantly different for the Glonass-only case; indeed, it was possible to compute the Glonass solution with Mi8 during less than half the session period. In general, for single point positioning without RAIM application, neither device seemed to prevail over the other in terms of performance.

### 5.4. Analysis of Position Performance, Applying RAIM Functionality

The main objective of this work was to analyze the performance of Xiaomi Mi8 GNSS in a signal-degraded environment, applying RAIM functionality; the same algorithm was also applied to the NVS receiver. The comparison between Xiaomi Mi8 and NVS with RAIM application is summarized in Table 5. Considering only the GPS measurements, Mi8 and NVS performances were very similar. Indeed, the horizontal RMS error was slightly smaller for NVS, while the vertical RMS error was slightly smaller for Mi8; conversely, the horizontal maximum error was smaller for Mi8, and the vertical one was smaller for NVS. Considering only the Glonass measurement, NVS performance clearly overcame that of Mi8 for all figure of merits; moreover, the NVS errors were referred to a significantly larger percentage of epochs (94.4% against 43.7% for Mi8). In the GPS/Glonass case, both NVS RMS and maximum errors were significantly smaller with respect to those of Mi8.

In Table 6, the same comparison is presented, but the errors are referred only to the reliable epochs of the considered configuration, according to the RAIM algorithm. The percentage of reliable epochs is reported in the last column, indicated as “reliable availability”. In this case, the performance of NVS overcame that of Mi8 in terms of RMS errors; the opposite happened for maximum errors, because the maximum errors for NVS happened in epochs not reliable for Mi8. In detail, considering only the GPS measurements, the horizontal RMS errors were 14.9 m for Mi8 and 10.2 m for NVS, the vertical RMS errors were 29.1 m for Mi8 and 26.8 m for NVS; moreover, the horizontal maximum errors were 129.1 m for Mi8 and 160.4 m for NVS, and the vertical maximum errors were 424.9 m for Mi8 and 455.0 m for NVS. The reliable availability was 74.2% for Mi8 and 79.4% for NVS. In Mi8, Glonass measurements were characterized by bad quality (as just highlighted in Table 3); in fact, the Glonass-only positioning was characterized by very large errors (horizontal and vertical RMS errors of 63.8 m and 117.7 m, respectively), and reliable availability was very low, 3.2%. This influenced also the GPS/Glonass positioning, which was degraded with respect to the GPS-only case. In NVS, Glonass showed better performance with respect to Mi8; in particular, the combined GPS/Glonass positioning provided similar performance to that of the GPS-only case, but with an increase of about 5% in reliable availability.

**Figure 5:**
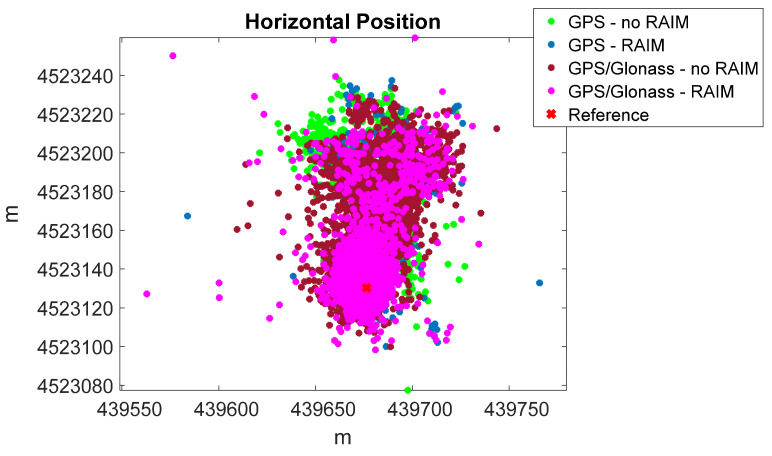
Horizontal position with Xiaomi Mi8, with and without RAIM application.

**Figure 6:**
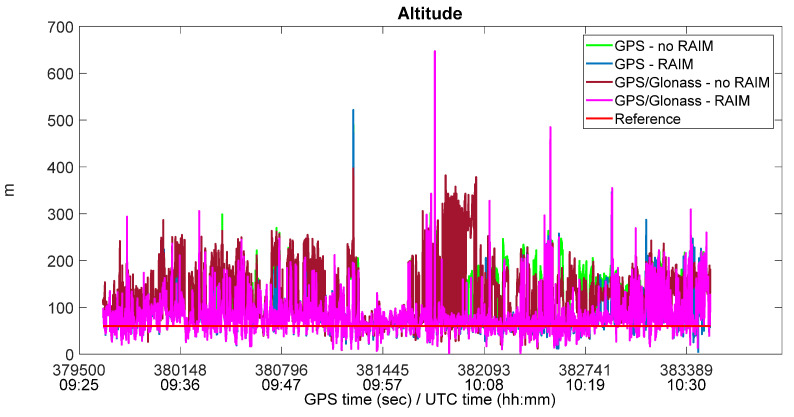
Vertical solution with Xiaomi Mi8, with and without RAIM application.

It should be highlighted that the application of the RAIM algorithm on Mi8 measurements is effective; indeed, as shown in Table 4 and Table 5, there was a significant improvement in positioning performance when RAIM functionality was active. For GPS-only, the RMS horizontal and vertical errors decreased by about 35% and 37%, respectively, with RAIM. For GPS/Glonass, the RMS horizontal and vertical errors decreased by about 47% and 52%, respectively, with RAIM. On the other hand, there were no significant improvements for Glonass-only, because the redundancy necessary for RAIM applicability was very rare during the session. The benefits of RAIM application on Mi8 measurements are also evident in Figure 5, where the horizontal positions obtained with GPS and GPS/Glonass measurements are shown. The green and blue dots represent GPS-only positions with and without RAIM, while the brown and magenta dots represent GPS/Glonass positions with and without RAIM. It is clear that when RAIM was active, the positions were more concentrated around the ground truth, indicated with a red cross in the Figure. Similar conclusions can be drawn from Figure 6, where the vertical solutions for the same configurations are shown, and the same color convention is adopted.

## 6. Conclusions

Since 2016, an increasing number of smartphones are able to provide raw GNSS measurements, allowing the implementation of advanced processing techniques and the development of new applications for mobiles. Several research studies highlighted the poor quality of measurements by smartphone chips, in case of multipath. In this research, the performance of a particular device, Xiaomi Mi8, was assessed in an urban environment, applying the RAIM technique to mitigate the effect of the multipath phenomenon. As a term of comparison, an NVS NV08C-CSM receiver, a low-cost high-sensitivity device connected to a patch antenna, was placed in the same location; the same algorithms were used for both devices. GPS and Glonass constellations were considered.

About 1 h of data were collected in a severe urban scenario, and the obtained results demonstrated the effectiveness of RAIM applied to smartphone GNSS chips; indeed, the RMS error decreased by about 35% in the GPS-only case, and by about 50% in the GPS/Glonass case. Considering only epochs indicated as reliable from RAIM, the GPS-only horizontal RMS error was about 10 m; taking into account the challenging scenario, this performance is very promising for the use of smartphone GNSS chips for applications requiring reliable and accurate positioning. From the comparison with the high-sensitivity receiver, it emerged that Xiaomi Mi8 is less performing, mainly because of its antenna.

## Figures and Tables

**Table 1:** Comparison between Xiaomi Mi8 and NVS satellite visibility.

Device	GNSS	Number of Available Satellites		
		Minimum	Average	Maximum
Xiaomi Mi8	GPS	2	6.6	10
	Glonass	0	3.6	6
	GPS/Glonass	3	10.2	15
NVS	GPS	6	9.0	10
	Glonass	3	5.3	7
	GPS/Glonass	9	14.2	17

**Table 2:** Comparison between Xiaomi Mi8 and NVS satellite geometry.

Device	GNSS	PDOP		
		Minimum	Average	Maximum
Xiaomi Mi8	GPS	1.5	3.6	16.6
	Glonass	2.2	7.7	25.2
	GPS/Glonass	1.3	2.6	10.9
NVS	GPS	1.5	2.0	5.6
	Glonass	1.7	4.8	25.3
	GPS/Glonass	1.1	1.5	2.9

**Table 3:** Comparison between Xiaomi Mi8 and NVS C/N0.

Device	GNSS	PDOP		
		Minimum	Average	Maximum
Xiaomi Mi8	GPS	11.0	33.2	43.0
	Glonass	10.0	30.7	43.0
NVS	GPS	13.0	42.7	55.0
	Glonass	12.0	39.6	53.0

**Table 4:** Comparison between Xiaomi Mi8 and NVS, without RAIM application.

Device	GNSS	RMS Error (m)		Maximum Error (m)		Solution Availability (%)
		Horizontal	Vertical	Horizontal	Vertical	
Xiaomi Mi8	GPS	39.4	80.1	114.6	461.5	99.7
	Glonass	104.4	198.7	819.8	859.7	43.7
	GPS/Glonass	42.1	94.7	106.2	336.7	99.9
NVS	GPS	45.4	101.3	111.3	256.0	100.0
	Glonass	53.5	160.0	562.3	552.5	94.4
	GPS/Glonass	40.8	111.4	97.4	261.4	100.0

**Table 5:** Comparison between Xiaomi Mi8 and NVS, with RAIM application (all available epochs).

Device	GNSS	RMS Error (m)		Maximum Error (m)		Solution Availability (%)
		Horizontal	Vertical	Horizontal	Vertical	
Xiaomi Mi8	GPS	25.5	50.7	156.1	461.5	99.7
	Glonass	104.4	198.7	819.9	859.7	43.7
	GPS/Glonass	22.9	45.6	156.1	587.1	99.9
NVS	GPS	21.1	53.5	160.4	455.0	100.0
	Glonass	48.0	144.3	562.3	552.5	94.4
	GPS/Glonass	16.1	38.3	146.5	434.4	100.0

**Table 6:** Comparison between Xiaomi Mi8 and NVS, with RAIM application (only reliable epochs).

Device	GNSS	RMS Error (m)		Maximum Error (m)		Reliable Availability (%)
		Horizontal	Vertical	Horizontal	Vertical	
Xiaomi Mi8	GPS	14.9	29.1	129.1	424.9	74.2
	Glonass	63.8	117.7	195.1	565.8	3.2
	GPS/Glonass	17.7	36.1	131.5	587.1	85.8
NVS	GPS	10.2	26.8	160.4	455.0	79.4
	Glonass	34.7	102.1	222.7	500.3	38.3
	GPS/Glonass	11.1	24.0	146.5	434.4	90.8
